# Underexpression of Specific Interferon Genes Is Associated with Poor Prognosis of Melanoma

**DOI:** 10.1371/journal.pone.0170025

**Published:** 2017-01-23

**Authors:** Aamir Zainulabadeen, Philip Yao, Habil Zare

**Affiliations:** 1 Department of Computer Science, Texas State University, San Marcos, Texas, United States of America; 2 Department of Computer Science, Princeton University, Princeton, New Jersey, United States of America; 3 Electrical Engineering and Computer Science, University of Michigan, Ann Arbor, Michigan, United States of America; Universidade de Sao Paulo, BRAZIL

## Abstract

Because the prognosis of melanoma is challenging and inaccurate when using current clinical approaches, clinicians are seeking more accurate molecular markers to improve risk models. Accordingly, we performed a survival analysis on 404 samples from The Cancer Genome Atlas (TCGA) cohort of skin cutaneous melanoma. Using our recently developed gene network model, we identified biological signatures that confidently predict the prognosis of melanoma (p-value < 10^−5^). Our model predicted 38 cases as low–risk and 54 cases as high–risk. The probability of surviving at least 5 years was 64% for low–risk and 14% for high–risk cases. In particular, we found that the overexpression of specific genes in the *mitotic cell cycle pathway* and the underexpression of specific genes in the *interferon pathway* are both associated with poor prognosis. We show that our predictive model assesses the risk more accurately than the traditional Clark staging method. Therefore, our model can help clinicians design treatment strategies more effectively. Furthermore, our findings shed light on the biology of melanoma and its prognosis. This is the first *in vivo* study that demonstrates the association between the *interferon pathway* and the prognosis of melanoma.

## Introduction

Cutaneous melanoma is a malignancy of melanocytes. It is the most common type of skin cancer. The American Cancer Society estimates that over 73,000 new cases were diagnosed in 2015 in the United States and about 10,000 deaths are caused by melanoma each year [1]. The prognosis of melanoma is highly variable [2]. For instance, the 5–year overall survival rate can be as high as 97% for stage I and as low as 3% for stage IV [3, 4]. Almost all common treatment options for melanoma, including surgery, chemotherapy, and radiation therapy, have harmful and severe side effects. Therefore, it is critical to identify patients who are not at a significant risk of metastasis and death due to the disease. The predictive power of clinical factors is limited [3, 5, 6] (e.g., staging based on the tumor size and the number of metastatic sentinel lymph nodes [7]), therefore clinicians are seeking more accurate molecular markers to improve risk models and to avoid unnecessary treatment of low-risk patients [8–10].

Gene expression profile signatures have useful information on the molecular status of cells and they can predict the prognosis of many cancers [11–14], including melanoma [10, 15–18]. For example, Onken *et al.* discovered a gene expression prognostic signature that significantly improved the classification of uveal melanoma compared to traditional staging [15]. That is, they showed that the deregulation of the *LEDA*, *FZD6*, and *ENPP2* genes predicts metastatic death (p-value < 10^−4^). In the follow–up studies, they extended their test to include 15 genes [19] and their extended test correctly classified 446 (97%) of the 459 studied cases into low–risk (i.e., at least 95% chance of 5–year metastasis–free survival) and high–risk (i.e., not more than 20% chance of 5–year metastasis–free survival) groups [20].

Recently, Gerami *et al.* performed a meta-analysis on several published genomic analyses of cutaneous melanoma tumors [15, 21–27]. Based on the gene ontology [28] of the frequently reported genes, they identified 28 discriminant genes including *BAP1, MGP, SPP1, CXCL14, CLCA2, S100A8, BTG1, SAP130, ARG1, KRT6B, GJA1, ID2, EIF1B, S100A9, CRABP2, KRT14, ROBO1, RBM23, TACSTD2, DSC1, SPRR1B, TRIM29, AQP3, TYRP1, PPL, LTA4H*, and *CST6* [4]. They used the expression of these genes to train a generalized linear mixed model with a radial basis function kernel. The resulting signature classified 268 primary cutaneous melanoma tumors into low-risk and high-risk groups, with 5–year disease–free survival rates of 97% and 31%, respectively [4]. This test is commercially available as a diagnostic tool called DecisionDx-Melanoma [29, 30], but it is not yet recommended by the National Comprehensive Cancer Network [10]. Its benefits to the patients must be confirmed using prospective clinical trials that include significantly larger cohorts with more representative metastatic characteristics [18, 31].

We hypothesized that there is room for significant improvement in the melanoma prognostic tests through rigid, unbiased, and comprehensive analysis of gene expression profiles [10, 32]. Accordingly, we applied a robust large-scale network analysis to the gene expression data of 404 samples from The Cancer Genome Atlas (TCGA) cohort of skin cutaneous melanoma [33]. The aim of our study was to identify the molecular signatures that predict the prognosis of melanoma and to segregate patients into low–, medium–, and high–risk groups. Our approach is based on co-expression network analysis, and we use *eigengenes* as informative prognostics signatures [34].

## Materials and Methods

### The TCGA Dataset

Several mRNA expression profiling datasets have been produced to study melanoma prognosis [10, 23, 27, 33, 35–40]. We used the TCGA2STAT package to download gene expression data from The Cancer Genome Atlas (TCGA) repository [41]. Specifically, we downloaded RNA-Seq data from the skin cutaneous melanoma cohort of 473 patients [33] and used RPKM values (i.e., reads per kilobase of transcript per million mapped reads) [42] as a measure of gene expression. We manually downloaded the corresponding clinical data, including: a) information regarding the last status of each case (i.e. whether the event of disease recurrence or progression occurred), b) the length of the disease–free time period (i.e., the time from the initial melanoma diagnosis until this event or until the last follow–up date if the event did not occur), and c) the Clark scale stage of the melanoma, which was determined using clinicopathological features such as the size, number, and location of metastases [43].

We computed the Spearman’s rank correlation between the disease–free time and gene expression [44]. Spearman’s rank correlation is more robust than Pearson correlation and it is the recommended approach for skewed distributions [45]. For instance, Mukaka showed that after removal of the outliers, the change in the Spearman’s correlation can be negligible unlike the Pearson correlation [46]. Consistent with the approach taken by other scholars [47], we used only the top third of genes (6,834) that were most correlated with the disease–free time in our network analysis. We considered any progressed or recurred tumor as *high risk* (*n* = 263). In the rest of the tumors, which were not reported to recur during the follow–up period, any case that had at least five years of follow–up data was considered *low-risk* (*n* = 33) [48–51]. All results presented here can be conveniently reproduced using our supplementary code (S4 File).

### Validation datasets

To confirm the findings that we obtained using the TCGA dataset, we validated them using two independent datasets. Specifically, we used the Leeds melanoma gene expression set 1 [39], which is publicly available through European Molecular Biology Laboratory–European Bioinformatics Institute (EMBL–EBI, accession number: E-MTAB-4725). For brevity, we refer to this dataset as LEED in this paper. This cohort comprises whole–genome mRNA expression of 204 primary melanoma tumors, which are measured using Illumina DASL HT12.4. Kolesnikov *et al.* normalized the gene expression values with quantile method after background correction. We manually downloaded the gene expression and clinical data from the EMBI–EBI ArrayExpress database (http://www.ebi.ac.uk/arrayexpress/experiments/E-MTAB-4725/) [52]. We used the “last follow up” (time) and “viability” (death event) columns from the clinical data.

We also used a similar cohort that was produced at the Lund University in 2015 [40]. This dataset is publicly available through EMBL–EBI (accession number: E-GEOD-65904) and also through Gene Expression Omnibus (GEO) (accession number: GSE65904) [53]. For brevity, we refer to this dataset as LUND in this paper. Cirenajwis *et al.* extracted total RNA from 214 fresh–frozen melanoma tumors and performed genome-wide expression profiling using Illumina Human HT-12V4.0 BeadChip arrays. We downloaded the gene expression data using GEOquery package (Version 2.40.0) [54]. We downloaded the corresponding clinical data from the EMBI–EBI database (http://www.ebi.ac.uk/arrayexpress/experiments/E-GEOD-65904/) and used the “disease specific survival” (time) and “disease specific death” (event) columns.

### Gene network analysis

We applied the weighted gene co-expression network analysis (WGCNA) (Version 1.51) to all 473 available samples to build a gene network and to cluster the genes into gene modules (clusters) [55]. Specifically, we used the calculate.beta function with the default parameters to infer that the soft-thresholding power for network construction was 7. We identified 13 gene modules using the wgcna.one.step function from the Pigengene package [34], which is a wrapper for the blockwiseModules function, with power = 7 and left the remaining arguments as defaults. WGCNA could not confidently assign 1,404 genes to any of the modules, because these genes had little correlation with the other genes. We call the set of these outlier genes Module 0.

### Computing eigengenes

An eigengene of a module is a weighted average of the expression of all the genes in that module. These weights are adjusted so that the loss of biological information is minimized [56, 57]. We used principal component analysis (PCA) to compute eigengenes. First, we balanced the number of high–risk and low–risk cases using oversampling so that both groups had comparable representatives in the analysis. Specifically, we repeated the data of each high–risk and low–risk case 6 times and 45 times, respectively. This approach provided us with 1,485 and 1,578 samples from each group, respectively. Oversampling was necessary for computing the eigengene of a module. Because an eigengene is the first principal component of the module, it would be biased towards the high–risk group, which has around eight times more samples than the low–risk group. Oversampling resolves this issue. Then, we applied the moduleEigengenes() function from the WGCNA package to the oversampled data. This function computed the first principal component of each module, which maximized the explained variance, thus ensuring a minimum loss of biological information. We used the project.eigen function from the Pigengene package (Version 0.99.23) to infer the values of eigengenes for all of the 473 samples in the TCGA, the 204 samples in the LEEDS, and the 214 samples in the LUNDS datasets (S1 File) [34].

### Survival analysis

We used the 14 inferred eigengenes as covariates (prognostic features), and we included only the 404 samples for which the final status and the survival time were available. We used the glmnet() function from the glmnet package (Version 2.0-5) [58] to perform a penalized Cox regression analysis [59, 60]. We set *α* = 1 to use the least absolute shrinkage and selection operator (Lasso) [61]. The Lasso, also known as *L*_1_ regularization, enforces most of the coefficients of the covariates (eigengenes) in the Cox proportional hazards model to be zero. Thereby, it identifies the modules that are the most associated with survival.

To evaluate the significance of the selected modules in predicting the survival time, we fitted an accelerated failure time (AFT) model to the selected eigengenes [62]. We used the survreg function from the survival package (Version 2.39-4) [63], set the Weibull distribution with scale = 1 as the baseline hazard function, and used the default values for the rest of the parameters. We used the fitted accelerated failure time model to predict the survival time of each sample. We chose two thresholds for the predicted values that maximized the precision of low– and high–risk predictions. The samples that had a predicted survival time between the two thresholds were considered medium–risk. We used the survfit function to obtain a Kaplan-Meier survival curve for each of the risk groups [64]. We used the survdiff function to test whether the survival curves that correspond to high–risk and low–risk groups differ significantly. This function computed the log-rank p-value of the corresponding Mantel-Haenszel test [65].

### Cross–validation

We performed 5–fold cross–validation to confirm that the selection of the modules by the penalized Cox regression is robust with respect to choosing the samples. We used all of the 14 eigengenes corresponding to the modules that were identified by our gene network analysis. We did not recompute the modules or eigengenes, instead, we repeated the penalized Cox regression model as follows. There were 404 samples for which the final status and the survival time were available. We randomly divided these 404 samples into five divisions. These divisions had an almost equal size and equal number of high–risk samples. We set aside one division and performed a penalized Cox regression analysis on the rest of the samples using *all* 14 eigengenes. We recorded the selected modules and repeated this procedure five times. We ran this experiment 10 times with different seeds and counted the frequency of the selected modules in each run (S2 File). On average, the three most frequent modules were selected 4.9, 4.8, and 4.2 times, respectively. In contrast, all other modules were selected 0.6 times or less, on average.

## Results

Using coexpression network analysis, we identified 13 modules of highly coexpressed genes. The size of thees modules ranges from 48 to 2,247 genes with a mean of 418, a median of 88, and a standard deviation of 629 (S1 Fig). We computed an eigengene for each module, which summarizes the biological information of the module into one value per sample. We used these eigengenes as biological signatures (features) to perform a survival analysis.

The penalized Cox regression consistently selected three modules as the most associated modules with disease–free survival (Methods). These three gene modules include the *outlier module* (with 1,404 genes, ME0), the ninth largest module (with 58 genes, ME9), and the twelfth largest module (with 52 genes, ME12) (S3 File). The outlier module consists of genes that have too small a correlation with other genes to be included in any of the modules. Hypergeometric tests revealed that the other two selected modules are associated with the *mitotic cell cycle* and the *interferon (IFN) pathway*, respectively [66] (S2 Fig). That is, 15 (29%) of the 58 genes in the larger module are members of the Reactome mitotic cell cycle pathway, which has 454 genes (adjusted p-value < 10^−9^) [67]. These genes include *AURKB, CCNE1, CDCA8, CDK4, CENPO, GINS2, H2AFZ, LIG1, PKMYT1, PLK1, PTTG1, SKA1, TUBA1B, TUBA1C,* and *TYMS*. According to the Cox model, overexpression of these genes is associated with poor prognosis, which is expected [68, 69].

The smaller module (ME12) has 52 genes. Interestingly, 16 (31%) of these genes are members of the Reactome interferon signaling pathway, which has 158 genes (adjusted p-value < 10^−21^). The genes in the overlap include *DDX58, EIF2AK2, GBP3, HERC5, IFIT1, IFIT2, IFIT3, OAS1, OAS2, OAS3, PSMB8, SP100, STAT1, UBE2L6, USP18,* and *XAF1*. Our model indicates that the relatively higher expression of these genes is associated with a good prognosis in melanoma. Our *in silico* overrepresentation analysis showed that *type I interferon signaling pathway* (Gene Ontology accession number: 60337 [28]), which includes 63 genes, is the biological processes that has a significant overlap with this gene set. Specifically, the overlap consists of 12 genes, which is 75 times more than expected (p-value = 10^−15^) [70]. This module is very stable with respect to selection of samples. To confirm this, we reconstructed the coexpression network 10 times using only 426 (90%) randomly selected samples. All of the resulting networks had a similar module, i.e., mean and median of the Jaccard [71] (Tanimoto [72]) similarity were 0.92 and 0.93, respectively.

**Fig 1:**
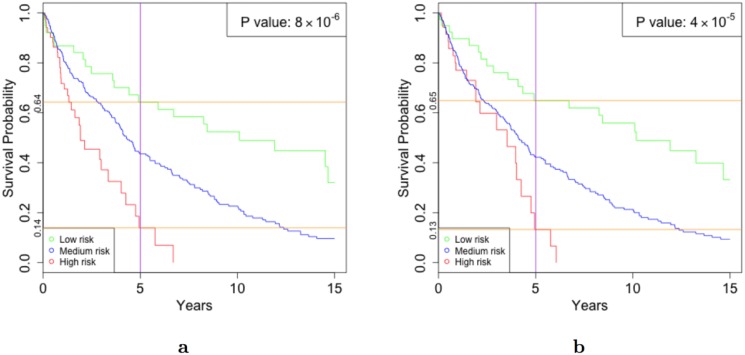
Kaplan–Meier survival curves. The p-values indicate that the difference between the low–risk group (green) and the high–risk group (red) is statistically significant. Using all the three modules, which are associated with the interferon pathway, mitotic cell cycle, and outliers; results in a better p-value (**a**) compared to a model without the interferon pathway (**b**). The orange horizontal lines indicate that both models have similar accuracies. However, including the interferon pathway improves the p-value, because more samples are classified in total (i.e., 38 low–risk plus 54 high–risk cases in (a), compared to 40 low–risk plus 35 high–risk cases in (b)).

To further validate the association of these three selected modules with melanoma prognosis, we fitted an accelerated failure time (AFT) model to the corresponding eigengenes, and classified the patients into low–, medium–, and high–risk groups (Methods) [62]. We compared the Kaplan-Meier (KM) curves of these groups [64] (Fig 1). Our AFT model predicted that 38 cases were low–risk. These cases had a significantly higher survival rate than the 54 cases that were predicted to be high–risk (log-rank p-value < 8 × 10^−6^ [73]). For instance, the probability of surviving for at least five years was 0.64 for low–risk and 0.14 for high–risk cases. Excluding the interferon module has a negative impact on the statistical power and the p-value (Fig 1). Specifically, while the number of cases in the predicted low–risk group does not increase dramatically (i.e., two cases, only 5% improvement), the number of predicted high–risk cases decreases considerably to 35 (i.e., 35% decline). That is, using the interferon pathway module, the model can identify more high–risk cases without sacrificing the accuracy.

As expected, the cases that were predicted to be high–risk had generally more advanced disease according to the traditional Clark scale stage of melanoma (Table 1). In particular, the majority of the high–risk cases were in stage IV or V (36 cases, 66%). In contrast, only 13 (35%) of the low–risk cases were in stage IV or V. Interestingly, 6 (46%) of these 13 cases were disease–free for more than eight years, which indicates that, for these cases, the Clark scale staging is less accurate than our predictions. Also, 7 cases had stage I, II, or III melanoma, but they were classified as high–risk by our model. Only 2 (29%) of these cases survived more than 2 years.

**Table 1:** The distribution of melanoma Clark stages in each risk group. Also, the percentage of each stage class in each risk group is shown. The low–risk group is enriched in patients at stages III and IV. The high–risk group is enriched in patients at stages IV and V.

Stage	I (%)	II (%)	III (%)	IV (%)	V (%)	Unknown (%)	Total
Low–risk	0 (0)	1 (3)	11 (29)	12 (32)	1 (3)	13 (34)	38
Medium–risk	4 (1)	16 (5)	54 (17)	105 (34)	37 (12)	96 (31)	312
High–risk	1 (2)	0 (0)	6 (11)	24 (44)	12 (22)	11 (20)	54
Total	5	17	71	141	50	120	404

To further validate the association between the interferon module and melanoma prognosis, we used the LEEDS and LUND gene expression datasets, which include 204 and 214 melanoma samples, respectively. We inferred the eigengenes and fitted an AFT model to the eigengenes to classify the samples into low–, medium–, and high–risk groups in each dataset (Methods). Similar to the analysis on TCGA dataset, we used the three eigengenes corresponding to the outlier, *mitotic cell cycle* and *interferon pathway* modules.

Our AFT model predicted 96 cases in the LUND dataset to be low–risk and 45 cases to be high–risk. The predicted low–risk group had a significantly higher survival rate than the high–risk group (log-rank p-value 2 × 10^−3^, S3a Fig). Excluding the *interferon pathway* module from the AFT model resulted in reducing the number of predicted low–risk to 49 cases and predicted high–risk to 25 cases, and also, a less significant p-value (3 × 10^−2^). That is, without the *interferon pathway*, the number of predicted low– and high–risk samples would be reduced by almost half leaving 67 (33%) more samples in the medium–risk group. This shows the necessity of the *interferon pathway* module in predicting the prognosis. The results in the LEEDS dataset followed a similar pattern. Specifically, excluding the *interferon pathway* module from the AFT model resulted in reducing the number of predicted low–risk samples from 34 cases to 31 cases while slightly decreasing the number of predicted high–risk samples from 29 to 28. Nevertheless, the log-rank p-value increased by almost two orders of magnitude, from 9 × 10^−6^ to 7 × 10^−4^, indicating that the prediction of survival is less accurate without the *interferon pathway* module (S3c and S3d Fig).

## Discussion

Predicting the prognosis of melanoma is clinically useful and important [10]. To date, most of the studies that aim at predicting melanoma survival based on gene expression have been limited in their number of genes, number of samples, or their follow–up time [4, 15–27, 30, 74]. We performed gene network analysis on 470 melanoma cases to extend the previous studies and to identify novel prognostic signatures.

This is the first study to show that the underexpression of specific genes from the interferon pathway in melanoma tissues is a sign of poor prognosis. The role of the interferon pathway in other cancers were studied by others [75–80]. In general, defects in interferon signaling results in dysfunction of the immune system [81]. However, its association with melanoma was previously shown only *in vitro* [81–84].

Interestingly, our interferon module has 17 genes in common with the 274 genes that Hoek *et al.* reported to be downregulated in melanoma cell lines [82]. This is a significant overlap (p-value of the hypergeometric test < 10^−19^). Similarly, the list of the top 25 genes that Critchley *et al.* reported to be differentially expressed in peripheral blood mononuclear cell (PBMC) samples of melanoma patients has a significant overlap with our interferon module (13 genes, p-value < 10^−27^). Compared to *in vitro* experiments, our analysis provides much stronger evidence for the role of the interferon pathway in melanoma, because our study is based on the survival analysis of a relatively large cohort of patients with an extended follow–up time. The total number of patients classified as low–risk or high–risk increases from 75 to 92 (a 23% improvement) when we include the interferon module in our predictive model. This improvement, as well as the decrease in p-value, indicate that the information in the interferon pathway is essential for predicting the prognosis.

However, functional studies will be needed to determine the mechanism and impact of the *interferon pathway* on melanoma prognosis. One challenge in designing such a study is possibly the relatively low number of samples that could be associated with the genes in the interferon module. For example, in our study on the 404 TCGA cases, including these genes in the predictive model led to classification of only 17 (4%) more cases. Therefore, a follow-up functional study most likely needs to investigate at least hundreds of samples in order to include a few samples that are associated with the *interferon pathway*.

The probability of surviving for at least five years is 0.64 and 0.14 for our predicted low–risk and high–risk groups, respectively. The accuracy of our predictive model is comparable with previous studies on skin cutaneous melanoma that were based on gene expression [10]. For instance, Sivendran *et al.* developed a log-rank Mantel–Cox test based on a 21-gene expression signature and ulceration [74]. Their test is less specific than ours in predicting long-term poor prognosis. That is, they tested 48 patients and reported that the probability of surviving for at least five years was 60% and 35% for their predicted low–risk and high–risk groups, respectively.

Gerami *et al.* reported that the DecisionDx-Melanoma test [29, 30] classified 268 primary cutaneous melanoma tumors into low-risk and high-risk groups with at least 5–year disease–free survival rates of 97% and 31%, respectively [4]. Their low–risk specificity is more than ours, and their high–risk specificity is less than ours. However, the reported assessment of the sensitivity and specificity of this test is controversial, because their cohort was not representative of the general primary melanoma patient population [10, 18]. Our results are not comparable with the results reported by Onken *et al.* [20], because they studied uveal melanoma, which is different from skin cutaneous melanoma [85].

One reason underlying the difficulty in assessing the survival rate of melanoma is the relatively high heterogeneity of genetic mutations, which results in subpopulations (clones) that are resistant to therapy [86–88]. Recently, Tirosh *et al.* used single-cell sequencing to show that distinct clones within a tumor can have different gene expression profiles, and therefore, can interact with their microenvironment differently [89]. Investigation of the genes in our interferon module and the corresponding signaling proteins may reveal how the interactions between malignant cells and their microenvironments is modulated. For instance, the relatively high expression of *type I interferon signaling pathway* genes in low–risk melanoma cases can be associated with antitumor response of the immune system [90]. Future work in this direction can leverage available techniques for 1) clonal decomposition based on genetic mutations [91–97], and 2) deconvolution of gene expression profiles into signatures that are specific to a cell-type or tumor clone [98–101].

One limitation of our predictive model for clinical use is the relatively high number of cases that were classified as medium–risk (312, 77%). This can be addressed by improving the predictive model in a follow–up study or, alternatively, by using another prognostic test for the medium–risk cases. Compared to other tests, our predictive model is based on a relatively large number of genes. This is a double-edged sword. The inclusion of a large number of genes makes our model robust with respect to random changes in the expression of one or several genes, which are common due to technical or biological noise. On the other hand, the large number of genes makes our test difficult to apply in clinical settings. This can be addressed by excluding the genes that have a relatively smaller contribution to the eigengenes. Specifically, a greedy algorithm can be used to exclude the genes that have a smaller absolute weight (loading) [102]. Further follow–up experiments on different datasets will be needed to show that such a modification does not affect the accuracy of the model and to ensure that too many genes will not be excluded. Nevertheless, follow-up studies can examine the therapeutic value of the genes identified in our study that have a relatively high contribution to the model, as well as their upstream genes (S3 File).

## Conclusion

We identified a specific set of genes in the interferon pathway that are underexpressed in high–risk melanoma. This biological signature, together with the overexpression of other genes in the mitotic cell cycle pathway, predicts the prognosis of melanoma with relatively high accuracy.

## Supporting Information

S1 File The eigengene values in the TCGA dataset useful for reproducing the results. (XLS)

S2 File The frequency of selected modules by 5–fold cross-validation of the penalized Cox model. (XLS)

S3 File The gene lists corresponding to the three selected modules. The gene symbol, Entrez ID, and the weight of each gene in the corresponding selected module is reported.
(XLS)

S4 File Supplementary code. Our results can be conveniently reproduced using these R scripts. Uncompress the tarball file, install the packages mentioned in the settings.R script, and then source the runall.R script. This code works on Unix (Linux or Mac OS X). Data will be downloaded from TCGA and the results will be saved in the current directory. A desktop computer with a 2.8 GHz CPU and 8 GB of memory will reproduce all results in less than an hour.
(ZIP)

S1 Fig The distribution of the size of modules. (PNG)

S2 Fig The overrepresentation analysis on the selected modules. (PNG)

S3 Fig The Kaplan–Meier survival curves for the validation datasets. Colors are similar to (Fig 1). In the LUND dataset, including the *interferon pathway* module results in better predictions of the survival time **(a)** with a more significant p-value of 2 × 10^−3^ compared to an AFT model that uses only two modules **(b)**. Similarly, in the LEEDS dataset, the model predicts the survival rate better when the *interferon pathway* module is included **(c)** compared to a model that uses only two modules **(d)**.
(PNG)
