# Supplementary figures and images for: Underexpression of Specific Interferon Genes Is Associated with Poor Prognosis of Melanoma
Source: PLoS One. 2017 Jan 23;12(1):e0170025. doi: 10.1371/journal.pone.0170025 (PMC5256985; doi:10.1371/journal.pone.0170025)

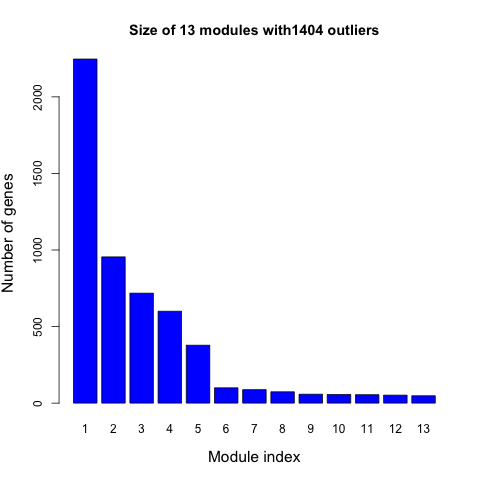

Supplement: S1 Fig — (PNG) [file pone.0170025.s005.png]

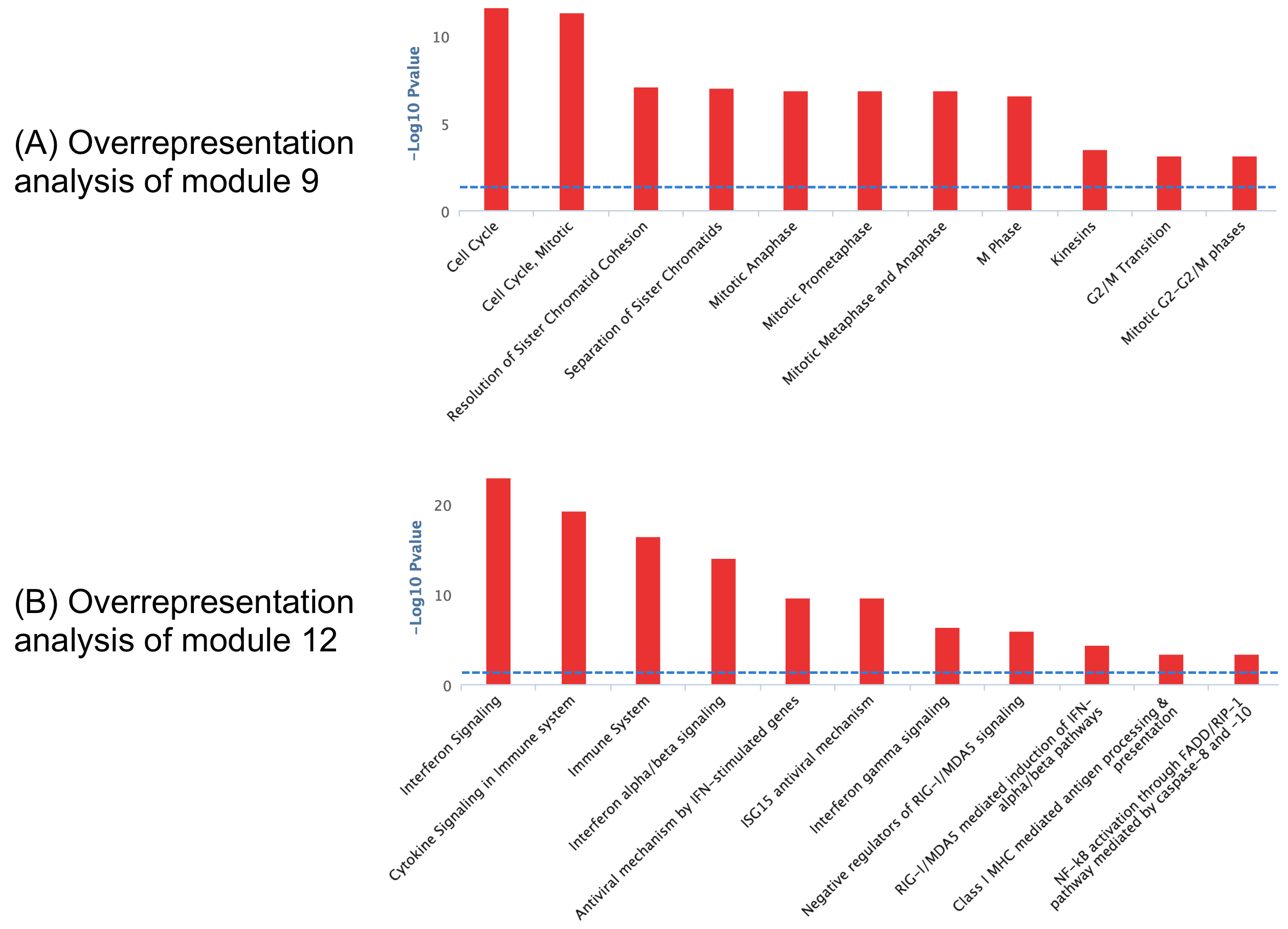

Supplement: S2 Fig — (PNG) [file pone.0170025.s006.png]

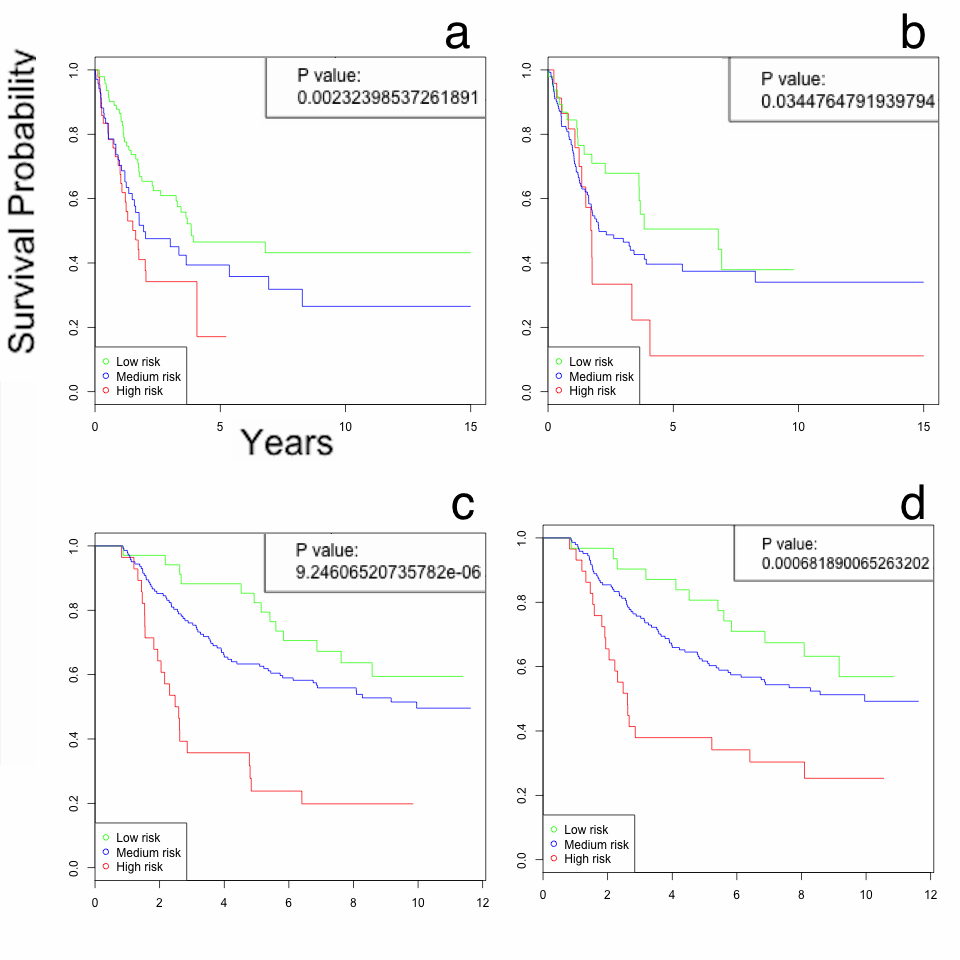

Supplement: S3 Fig — Colors are similar to (Fig 1). In the LUND dataset, including the interferon pathway module results in better predictions of the survival time (a) with a more significant p-value of 2 × 10^−3^ compared to an AFT model that uses only two modules (b). Similarly, in the LEEDS dataset, the model predicts the survival rate better when the interferon pathway module is included (c) compared to a model that uses only two modules (d). (PNG) [file pone.0170025.s007.png]
